# First Comparative Evaluation of Short-Chain Fatty Acids and Vitamin-K-Dependent Proteins Levels in Mother–Newborn Pairs at Birth

**DOI:** 10.3390/life13030847

**Published:** 2023-03-21

**Authors:** Tamás Ilyés, Marius Pop, Mihai Surcel, Daria M. Pop, Răzvan Rusu, Ciprian N. Silaghi, Gabriela C. Zaharie, Alexandra M. Crăciun

**Affiliations:** 1 Department of Molecular Sciences, University of Medicine and Pharmacy “Iuliu Hațieganu”, 400012 Cluj-Napoca, Romania; 2 1st Obstetrics and Gynecology Department, University of Medicine and Pharmacy “Iuliu Hațieganu”, 400012 Cluj-Napoca, Romania; 3 2nd Obstetrics and Gynecology Department, University of Medicine and Pharmacy “Iuliu Hațieganu”, 400012 Cluj-Napoca, Romania; 4 Department of Neonatology, University of Medicine and Pharmacy “Iuliu Hațieganu”, 400012 Cluj-Napoca, Romania

**Keywords:** short-chain fatty acids, vitamin-K-dependent proteins, matrix Gla protein, osteocalcin, Gla-rich protein, PIVKA, newborn, mother

## Abstract

Background: The interplay between vitamin K (vitK) (as carboxylation cofactor, partially produced by the gut microbiota) and short-chain fatty acids (SCFAs), the end-product of fiber fermentation in the gut, has never been assessed in mother–newborn pairs, although newborns are considered vitK deficient and with sterile gut. Methods: We collected venous blood from 45 healthy mothers with uncomplicated term pregnancies and umbilical cord blood from their newborns at birth. The concentrations of total SCFAs and hepatic/extra-hepatic vitK-dependent proteins (VKDPs), as proxies of vitK status were assayed: undercarboxylated and total matrix Gla protein (ucMGP and tMGP), undercarboxylated osteocalcin (ucOC), undercarboxylated Gla-rich protein (ucGRP), and protein induced by vitK absence II (PIVKA-II). Results: We found significantly higher ucOC (18.6-fold), ucMGP (9.2-fold), and PIVKA-II (5.6-fold) levels in newborns, while tMGP (5.1-fold) and SCFAs (2.4-fold) were higher in mothers, and ucGRP was insignificantly modified. In mother–newborn pairs, only ucGRP (r = 0.746, *p* < 0.01) and SCFAs (r = 0.428, *p* = 0.01) levels were correlated. **Conclusions:** We report for the first time the presence of SCFAs in humans at birth, probably transferred through the placenta to the fetus. The increased circulating undercarboxylated VKDPSs in newborns revealed a higher vitamin K deficiency at the extrahepatic level compared to liver VKDPs.

## 1. Introduction

### 1.1. The Placenta

The placenta is a crucial but transient organ in pregnancy, developed from fetal tissue, as an interface between the circulation of the mother and the fetus.

The placenta has two sides, a fetal and a maternal side. The circulation of the mother and the fetus do not come in direct contact with each other, as there is a thin layer of cells forming a barrier between the two. This thin layer is initially a double layer made up of cylindrical cells that merge to form a single layer called syncytiotrophoblast later during the pregnancy. These cells form chorionic villi that contain fetal capillaries and facilitate the exchanges between the maternal and fetal circulation [1]. In addition to its organization into a villous structure, the maternal side of the cells of the syncytiotrophoblast also present an array of microvilli that further increase the surface area available for transfer [2].

The syncytiotrophoblast is a continuous cell layer with no intracellular junctions. Thus, all transport must take place through the apical and basal membranes of the syncytiotrophoblastic cells. The exact nature of the transplacental transport remains poorly understood, although there are numerous postulations regarding the mechanisms by which it takes place [1]. It is accepted that trans-placental transport falls into one of three categories: passive diffusion, transporter protein mediated transfer, or endo-/exocytosis [1].

Diffusion is the main pathway by which blood–gas exchange takes place through the placenta. Even though the placental barrier thickness decreases as the pregnancy progresses, the rate of oxygen transfer remains relatively constant [3]. The diffusion of charged molecules is dependent on the electrical potential difference between the two circulations. There is a small potential difference across the human placenta, and as the pregnancy advances, it decreases even further [4,5]. While the diffusion of small liposoluble molecules occurs rapidly and is mainly dependent on blood-flow rate, the passive diffusion of hydro-soluble molecules is limited and is more dependent on transporter proteins [1].

The protein-mediated transport of molecules is of two types: facilitated diffusion (according to a concentration gradient) and active transportation (against a concentration gradient). An example of a molecule transported through facilitated diffusion through the placenta is glucose, via the GLUT family of transporter proteins [6]. The main advantage of protein-mediated transport compared to passive diffusion is the ability to maintain a relatively constant rate of transfer (through increased or decreased protein expression) in the case of physiological or pathological modifications to the transport surface [1].

**Figure 1:**
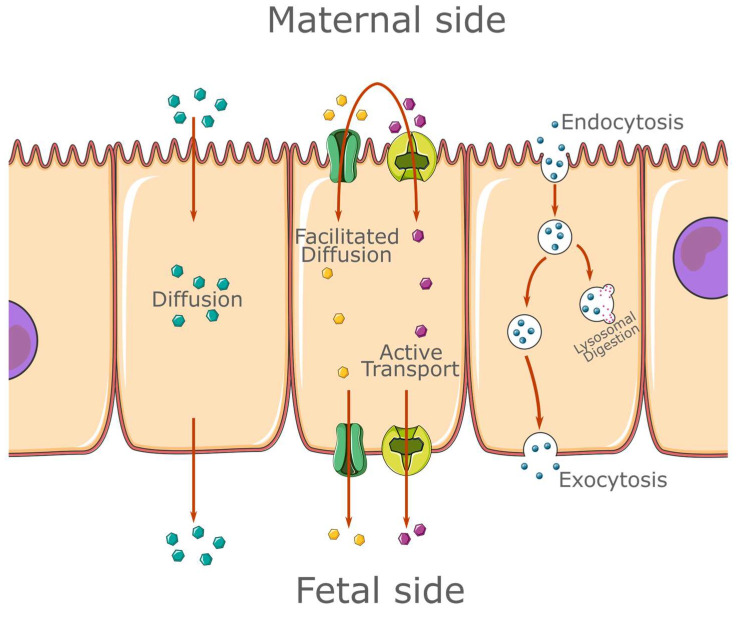
Summary of the trans-cellular transport mechanisms present in the syncytiotrophoblast. The figure was partly generated using Servier Medical Art, provided by Servier, licensed under a Creative Commons Attribution 3.0 unported license; https://smart.servier.com/ (accessed on 2 November 2022).

Endocytosis vesicles may fuse with lysosomes with the content being digested, or they may pass undisturbed through the cell [7,8]. These processes are summarized in Figure 1.

### 1.2. Vitamin-K-Dependent Proteins

Vitamin K has a number of structurally different vitamers, generally divided into two large groups: phylloquinones (vitamin K_1_) and menaquinones (MK) [9]. Both vitamin K_1_ and MK serve as cofactors in the carboxylation reaction of glutamic acid residues from proteins rich in them, also known as vitamin-K-dependent proteins (VKDPs) [10]. Carboxylation of these glutamic acid residues produces gamma carboxy glutamic acid (Gla), which enables VKDPs to bind calcium [11]. Human and animal requirements for vitamin K are difficult to establish because part of vitamin K is provided by food, and part is produced through biosynthesis by microorganisms in the digestive tract.

Vitamin K_1_ are present mainly in leafy green vegetables, the main vitamin K_1_ source of the human body being plant-based foodstuffs [12].

On the other hand, MK are found in meat and fermented dairy and cereal foodstuffs (curd cheese, natto, sauerkraut) but are also produced by the gut microbiota metabolism [13]. Fecal MK concentration has been found to vary according to microbiota composition [14].

The placenta also acts as a barrier for vitamin K, which leads to a ratio of vitamin K levels of about 30:1 between the maternal blood and cord blood of the fetus [15].

The most well-known hepatic VKDPs are the coagulation factors such as protein induced by vitamin K absence II (PIVKA-II), the undercarboxylated form of prothrombin, which is used to establish the vitamin K deficiency at the hepatic level. In comparison with hepatic VKDPs, there are many other VKDPs that are not produced in the liver (known as extra-hepatic VKDPs), the majority of them being involved in bone health and in preventing vascular calcification, which require higher amounts of vitamin K for their carboxylation [16]. Matrix Gla protein (MGP), osteocalcin (OC), growth arrest specific protein 6 (Gas6), and Gla rich protein (GRP) are some of the most studied extra-hepatic VKDPs, their carboxylation status depending on extra-hepatic vitamin K levels [17].

### 1.3. Short-Chain Fatty Acids

Short-chain fatty acids (SCFA) are carboxylic acids with up to five carbon atoms [17]. Methanolate, acetate, propionate, butyrate, and valerate are all considered SCFAs, but the majority of studies generally focus on acetate, propionate, and butyrate, as these are produced in the highest quantities in the gut. The main source of SCFAs in the body is through the fermentation of indigestible dietary fibers by the gut microbiota.

Short-chain fatty acids are absorbed from the large intestine in variable amounts and have multiple roles in the human body such as increasing leptin production, either directly or through two G-protein coupled receptors, GPR-41 and GPR-43, for which they are ligands [18,19,20,21,22].

Even if SCFAs have been detected in the meconium of newborns, only acetate and propionate were identified in significant quantities, while butyrate and valerate were absent [23]. Due to only acetate and propionate being present in meconium, this is either the result of inoculation of the newborn through contact outside the womb, or is the result of a yet unidentified production mechanism other than the gut microbiota.

The presence of SCFAs in the fetal circulation has only been identified in pigs [24] but not in humans. Because newborns at birth are considered sterile, the presence of SCFAs in their circulation could be the result of transfer from the mother.

### 1.4. Microbiota

Even if the fetus, up to the moment of birth, has long been considered as being completely sterile [25], recent studies have shown the presence of small quantities of bacteria in the amniotic fluid, the umbilical cord, as well as the placenta [23,26]. Furthermore, species as *Enterococcus* and *Staphylococcus* have been identified in the meconium [27]. The inoculation of these structures appears to be the result of ascension of microbes from the vaginal microbiota and/or of hematogenous dissemination. A concrete source of these microbes has yet to be identified [26].

Studies that have presented evidence of microbial presence in structures previously considered sterile, due to using very sensitive quantification techniques such as polymerase-chain reaction to identify bacterial DNA, could neither exclude accidental contamination nor prove unequivocal colonization in these structures [23]. Consequently, the question still remains whether the fetus is actually completely sterile, but if there is an existing fetal microbiota, it is not present in a high enough concentration to influence the presence of bacterial metabolites in the fetus. This suggests that any substances produced by bacteria probably originate from the mother and pass through the placenta to the fetus.

Considering that the placenta is a barrier between the mother and fetus for vitamin K originating from either the gut microbiota or from dietary intake, we evaluated the hepatic and extra-hepatic levels of undercarboxylated VKDPs as a result of vitamin K deficiency in newborns. Since the colon of the newborn is considered sterile, one of our goals was to assess the total SCFA concentration for the first time in mothers and umbilical cord blood of their newborns, hypothesizing the possibility of placental transportation of SCFAs.

## 2. Materials and Methods

### 2.1. Study Design

We included 45 mothers and their newborns from the No. 1 and No. 2 obstetrics and gynecology clinics of County Emergency Clinical Hospital of Cluj-Napoca, Romania. All included mothers gave their informed consent to participate in the study. All births took place between October and December of 2020, and all newborns were born at term.

Mothers admitted for childbirth, either via caesarian section or natural birth, were included in this study. Both smoking and non-smoking mothers were included. Only normally evolved pregnancies were taken into consideration.

Mothers who did not give their informed consent for participation in the study and cases in which either the mother or the child suffered severe complications related to childbirth were excluded. Newborns with an APGAR score less than 9 as well as their mothers were also excluded. Cases in which the mothers received peri-partum antibiotic or anticoagulant treatment were not included in the study.

### 2.2. Sample Collection and Storage

Venous blood samples were obtained from mothers immediately post-partum and simultaneously with the one collected from the umbilical cord of their newborns after cord clamping. The blood samples were collected into plain, additive-free vacuum tubes. After 30 min at room temperature, the samples were centrifuged at 3000× *g* for 10 min. The separated serum was aliquoted into sterile 1.5 mL microcentrifuge tubes and subsequently stored at −80 °C until analysis.

Anthropometric data were collected from patient observation files.

### 2.3. Sample Analysis

Total SCFAs, undercarboxylated osteocalcin (ucOC), undercarboxylated Gla-rich protein (ucGRP), PIVKA-II, total matrix Gla protein (tMGP), undercarboxylated matrix Gla protein (ucMGP), and vitamin D (vitD) concentrations were assayed using commercial enzyme-linked immunosorbent assay (ELISA) kits (MyBioSource^®^, San Diego, CA, USA) following the manufacturer’s protocols. After processing, the ELISA plates were read by an automated ELISA plate reader (Organon Teknika 230 s, Oss, The Netherlands).

Total calcium (Ca), glucose (Glu), triglycerides (TG), total cholesterol (TC), iron (Fe), magnesium (Mg), and phosphorous (P) levels were measured by conventional methods using an automated biochemistry analyzer (Mindray BS-480, Shenzhen Mindray Bio-Medical Electronics Co., Shenzhen, China). For all analyses, intra- and inter-assay coefficients of variation were under 10%.

### 2.4. Statistical Analysis

Statistical analysis of the gathered data was carried out using RStudio Desktop (RStudio© PBC v1.4.1106, Boston, MA, USA). A value of *p* ≤ 0.05 was considered statistically significant.

Analysis for the normality of quantitative data distribution was conducted using the Shapiro–Wilk test. Normally distributed data were presented as mean ± standard deviation, while non-Gaussian distributed data were presented as median (minimum–maximum). Qualitative data were expressed as frequencies. The correlation between quantitative data was analyzed using Pearson’s R coefficient or Spearman’s coefficient according to the normality of the data. Comparison of means and medians was accordingly carried out using either Student’s *t*-test or the Mann–Whitney U test.

## 3. Results

The total number of subjects included in this study was 90 (45 mothers and their newborns). The characteristics of the study participants are presented in Table 1 and Table 2. Table 2 also contains the comparison of the means and medians between mothers and newborns for parameters that were measured in both.

Parameters with significant positive correlations between mothers and newborns are presented in Table 3.

Total maternal cholesterol was significantly negatively correlated with newborn ucOC (r = −0.378, *p* < 0.05), maternal Ca with newborn PIVKA-II (r = −0.361, *p* < 0.05), maternal PIVKA-II with newborn Fe (r = −0.5, *p* < 0.05) and maternal BMI with newborn Mg (r = −0.350, *p* < 0.05).

Other parameters did not present any statistically significant correlations between mothers and newborns.

## 4. Discussion

The results obtained in this study in part are sustained by previous research and raise a number of hypotheses regarding the nature of the mother–fetus placental transfer of different compounds.

There was a marked difference between the levels of certain analytes between mothers and newborns. The levels of PIVKA-II were over five times higher in the newborns compared to the mothers. The levels of tMGP were, on the other hand, over five times higher in the maternal serum, while ucMGP had an opposite trend, being over nine times as high in newborns as in their mothers. Importantly, the placenta is considered a barrier for vitamin K between the mother and fetus [15]. This suggests the possibility that newborns present a constant, physiological deficit of vitamin K, and for certain VKDPs, the carboxylation reaction happens in mothers, but the carboxylated form of the VKDPs cannot traverse the placental barrier or does so only in minute quantities. Another possible explanation is that the various tissue carboxylases are not yet sufficiently mature in newborns [28]. It is known that vitamin K, as all liposoluble vitamins, is transported via facilitated diffusion through the syncytiotrophoblast and that this transport is relatively poor [15,29]. This confirms the fact that PIVKA-II is much higher in the serum of the newborns [30]. It would also explain why ucMGP is higher in the newborns, while tMGP (which takes into account the carboxylated conformations as well) is higher in the maternal circulation. The newborns simply have much less vitamin K than the mothers; thus, vitamin K deficiency bleeding (VKDB) in the newborn could not be ascribed to the immaturity of the liver, which forms insufficient coagulation factors, but is instead caused primarily by a vitamin K deficiency. Human milk is also a poor source of vitamin K. Breastfed infants are more susceptible to VKDB than those fed otherwise [28].

An interesting finding that provides a counter argument to the above is a strong positive correlation of vitamin D (another liposoluble vitamin) levels between mothers and newborns, along with the fact that vitamin D levels in the newborns were double that of the mothers, echoing the results of previous studies [31]. At first glance, this would suggest that the transport of vitamin D through the placenta is actually a very effective one. However, this is countered by the fact that, if these levels were the result of trans-placental transport, the levels should not be higher in the newborn than the mothers. More plausible explanations for this are either that vitamin D is actually transported as one of its precursors and then converted to the active form by the fetus, or that vitamin D is actually transported via active transportation against the gradient and is present in higher concentration in the fetus, because it is required for sustained growth. The latter is supported by the fact that ucOC levels were also higher in newborns, suggesting that ucOC is the result of vitamin D stimulatory effects on osteoblasts [28].

Regarding lipid metabolism and transfer, cholesterol levels were four times lower and triglycerides were eight times lower in newborns than their mothers, suggesting a poor transfer of lipids through placenta or poor endogenous cholesterol and triglycerides synthesis in newborns. Moreover, this large difference in lipid levels may result from food intake of mothers within 5–12 h before labor. On the other hand, vitamin D levels were two-fold higher in newborns than in their mothers, which was associated with significantly higher levels of calcium, phosphorus, and magnesium in newborns. Iron was also almost double in newborns compared to their mothers, suggesting that for oligoelements, vitamin D may play a role in the trans-placental transport by stimulating the fetus’ uptake of ions (see Table 2).

While previous studies reported PIVKA-II and OC levels in newborns [32,33] other extrahepatic undercarboxylated VKDPs were not taken into account, and neither was the degree of their change in newborns. We can hypothesize that vitamin K deficiency is reflected in the undercarboxylation of extrahepatic Gla proteins more than hepatic proteins.

An alternative hypothesis for the above-mentioned discrepancy in undercaboxylated VKDPs is that trans-placental differences in observed concentrations are influenced by the number of glutamic acid residues. This is sustained by the fact that the differences in concentrations between mothers and newborns coincide with the number of glutamic acid residues to be carboxylated in each VKDP: GRP contains 16 residues [34], followed by PIVKA-II with 10 [35], MGP with 5 [36], and OC with 3 [37]. This would suggest that the trans-placental transport of VKDPs is inversely proportional to the number of these residues present in each molecule. Confirmation of this hypothesis, however, will require studies that investigate the transport proteins responsible for trans-placental VKDPs transfer, as well as assaying the carboxylated and total VKDP concentrations for each protein.

While PIVKA-II and ucMGP both had differences between mothers and newborns, the same does not apply to GRP as well. In the case of ucGRP, there was no statistically significant difference between the concentrations in mothers compared to newborns. Moreover, there was a very strong positive correlation between the maternal and newborn levels of ucGRP. A possible explanation is that compared to the other VKDPs, GRP is actually transported in its undercarboxylated conformation through the placenta. This hypothesis is also sustained by the very strong positive correlation found in ucGRP between the maternal and newborn levels.

One of the weaknesses of this study was that only the undercarboxylated conformations of VKDPs were measured. This study assessed the undercarboxylated VKDPs, because the aim was to evaluate the extent of vitamin K deficiency in newborns. Further studies should measure the carboxylated VKDPs as well, in order to provide more insight into the carboxylation status in the newborn, as well as the ratio between the carboxylated and undercaboxylated conformations.

Because the fetus is considered sterile or near-sterile [23,25,26], any SCFA present in the fetal circulation would have a high probability of originating from the mother. The present study reports for the first time total SCFAs in newborn umbilical cord blood, which were less than half of their mothers. Because SCFAs can, theoretically, only originate in significant quantities in the mother’s gut microbiota, and because maternal and fetal SCFAs levels presented a strong positive correlation, it can be postulated that the newborn’s SCFAs are transferred from the mother through the placenta. This was also postulated in a previous study carried out in pigs [24].

Because the levels of SCFAs are higher in the mother’s circulation compared to the newborns, we assume that the transport takes place according to the concentration gradient. However, the SCFAs of the mothers are double those of the newborns, so even if this transfer appears to take place passively, it is still not very efficient. The human placenta was shown to be readily permeable to molecules between 1350–5200 Daltons [38,39]. Because SCFAs are much smaller and are hydro-soluble molecules, one possible mechanism is facilitated diffusion. The trans-placental transfer of SCFAs has yet to be studied and validated by further studies, especially by selectively comparing the amounts of different SCFAs.

This study assayed only total SCFAs in serum. In order to gain more insight into circulating SCFAs in newborns, future studies should determine each SCFA separately via gas chromatography coupled with mass spectrometry (GC-MS) or high-performance liquid chromatography coupled with MS (HPLC-MS). It would also be beneficial for future research to concomitantly determine the concentration of each individual SCFA from meconium, urine, umbilical cord blood, and maternal blood [40]. Such an approach would clarify the exact source of the SCFAs present in the newborn and the mechanism of the trans-placental trasport.

## 5. Conclusions

This study analyzed for the first time the trans-placental transfer of SCFAs, as well as the relationship between VKDPs and total SCFAs in serum as proxies for the maternal gut microbiota. Newborns were found to have a deficit of vitamin K, proven by the presence in higher concentrations of all undercarboxylated VKDP with higher concentrations of extra-hepatic compared to hepatic VKDPs. Therefore, in newborns at term compared with their mothers, ucOC and ucMGP, as extrahepatic VKDPs, are the most sensitive to vitamin K deficiency, in comparison with ucGRP or the hepatic PIVKA-II.

We report, for the first time, the presence of SCFAs in the umbilical cord blood and the correlation of SCFAs levels between mothers and their newborns at birth, raising the possibility of further research to focus on revealing the mechanism by which SCFAs are transferred from the mother to the fetus in utero.

## Figures and Tables

**Table 1:** Characteristics of study lot: quantitative morphometric variables.

Variable	Mean ± SD Median (Min–Max)	
	Mothers (n = 45)	Newborns (n = 45)
Age (years)	29.93 ± 4.65	N/A
Weight (kg)	75.44 ± 10.39	N/A
Height (cm)	164.93 ± 5.09	N/A
BMI (kg/m^2^)	27.7 ± 3.33	N/A
Gestational age (weeks)	N/A	40 (37–41)
Birth weight (g)	N/A	3421.95 ± 524.05
Birth length (cm)	N/A	53.68 ± 2.76

Abbreviations: N/A, not applicable; BMI, body mass index.

**Table 2:** Characteristics of study lot: quantitative analytical variables. Comparison of means and medians between mothers and newborns, where applicable.

Variable	Mean ± SD Median (Min–Max)		Magnitude of Difference (Newborns vs. Mothers)	*p*
	Mothers (n = 45)	Newborns (n = 45)		
SCFAs (ng/mL)	0.36 (0.02–1.45)	0.15 (0.04–1.73)	−2.4 ×	<0.05 *
tMGP (ng/mL)	137.5 (55.3–193.75)	26.84 (9.45–149.24)	−5.12 ×	<0.01 *
ucOC (ng/mL)	0.95 (0.06–3.86)	17.63 ± 8.09	+18.6 ×	<0.01 *
ucMGP (ng/mL)	11.54 (1.67–120.97)	106.64 (5.3–606.14)	+9.24 ×	<0.01 *
PIVKA-II (ng/mL)	14.45 (5.55–31.65)	80.7 (7.14–124.6)	+5.6 ×	<0.01 *
ucGRP (pg/mL)	9.81 (1.52–137.82)	11.82 (1.15–185.57)	N/A	0.38
vitD (ng/mL)	9.96 ± 5.41	22.9 (11–51.5)	+2.29 ×	<0.01 *
Glu (mg/dL)	68.34 ± 24.96	66.9 (3.4–125)	N/A	0.95
TG (mg/dL)	242.73 ± 68.03	30 (8.1–246.8)	−8.1 ×	<0.01 *
TC (mg/dL)	265.4 (186.1–451.6)	63.1 (25.9–263.5)	−4.2 ×	<0.01 *
Ca (mg/dL)	9.08 ± 0.31	10.72 (8.34–11.94)	+1.18 ×	<0.01 *
Mg (mg/dL)	1.84 ± 0.13	1.91 ± 0.19	+1.03 ×	<0.05 *
Fe (μg/dL)	72 (21–190)	139.33 ± 45.83	+1.93 ×	<0.01 *
P (mg/dL)	3.31 (2.07–6.25)	5.73 (2.43–2.06)	+1.73 ×	<0.01 *

Abbreviations: N/A, not applicable; SCFAs, total short-chain fatty acids; ucOC, undercarboxylated osteocalcin; ucGRP, undercarboxylated Gla-rich protein; PIVKA-II, protein induced by vitamin K absence II; tMGP, total matrix Gla protein; ucMGP, undercarboxylated matrix Gla protein; vitD, vitamin D; Ca, calcium; Glu, glucose; TG, triglycerides; TC, total cholesterol; Fe, iron; Mg, magnesium; P, phosphorus; ×, times. Statistically significant differences are in bold with *.

**Table 3:** Parameters with statistically significant positive correlations between mothers and newborns.

Parameters		Correlation Coefficient	*p*
Mothers	Newborns		
Height	ucMGP	0.372	<0.05
SCFAs	Gestational age	0.386	<0.05
TG	Weight	0.335	<0.05
tMGP	Birth length	0.398	<0.05
tMGP	PIVKA-II	0.339	<0.05
**SCFAs**	**SCFAs**	**0.428**	<0.05 *
SCFAs	ucGRP	0.326	<0.05
ucGRP	SCFAs	0.414	<0.05
**ucGRP**	**ucGRP**	**0.746**	<0.01 *
ucGRP	tMGP	0.338	<0.05
ucGRP	TC	0.391	<0.05
Glu	ucOC	0.373	<0.05
**Glu**	**Glu**	**0.337**	<0.05 *
**VitD**	**VitD**	**0.545**	<0.01 *
P	TC	0.386	<0.05
**Mg**	**Mg**	**0.336**	<0.05 *
ucOC	P	0.436	<0.05

Abbreviations: SCFAs, short-chain fatty acids; ucMGP, undercarboxylated matrix Gla protein; TG, triglycerides; tMGP, total matrix Gla protein; TC, total cholesterol; PIVKA-II, protein induced by vitamin K absence II; ucGRP, unercarboxylated Gla rich protein; Glu, glucose; ucOC, undercarboxylated osteocalcin; VitD, vitamin D; P, phosphorus; Mg, magnesium. Correlation coefficient: 0–0.3, weak correlation; 0.3–0.4, moderate correlation; 0.4–0.7, strong correlation; 0.7–1, strong correlation. Correlations between the same parameters for both mothers and newborns are in bold with *.

## Data Availability

Not applicable.
